# Circadian Rhythm and Physical Fatigue Separately Influence Cognitive and Physical Performance in Amateur Athletes

**DOI:** 10.3390/jfmk9040227

**Published:** 2024-11-08

**Authors:** Panagiota Karanika, Philip Gallardo, Themistoklis Tsatalas, Giannis Giakas, Panagiotis V. Tsaklis

**Affiliations:** 1 ErgoMech-Lab, Department of Physical Education and Sport Science, University of Thessaly, 42100 Trikala, Greece; pkaranik@uth.gr (P.K.); pgallardo@uth.gr (P.G.); ttsatalas@uth.gr (T.T.); 2 Department Molecular Medicine and Surgery, Growth and Metabolism, Karolinska Institute, 171 77 Solna, Sweden; 3 Center of Orthopaedics and Regenerative Medicine, Center for Interdisciplinary Research and Innovation, Aristotle University Thessaloniki, 54124 Thessaloniki, Greece

**Keywords:** circadian variations, muscular fatigue, reaction time, visual memory, strength performance

## Abstract

Background/Objectives: Circadian rhythm (CR) influences various physiological functions, including physical and cognitive performance, which fluctuate throughout the day. The present study aimed to investigate the combined and separate effects of CR and physical fatigue on cognitive and physical performance. Methods: A sample of 18 amateur athletes was subjected to a series of tests at three different times of the day: morning, afternoon, and evening. Fatigue was induced following an isokinetic concentric exercise combined with a 20 min treadmill run, followed by assessments of selected physical and cognitive tasks. Results: A repeated measure ANOVA did not reveal an interaction between CR and fatigue in cognitive performance (*p* > 0.05). However, a significant main effect of fatigue was observed in visual reaction time (VisRT) across all three timepoints. Moreover, peak torque (PT) and the peak torque fatigue index (PTFI) showed significant differences between the three times of the day, peaking in the evening. Conclusions: Although we found no interaction between CR and the physical fatigue state on selected cognitive parameters at the three times of the day, a separate effect of fatigue on cognitive performance was identified. Additionally, physical parameters exhibited peak values occurring in the evening hours. Future research should further explore underlying mechanisms that potentially influence cognitive performance at different times of the day.

## 1. Introduction

Circadian rhythm (CR) refers to organisms’ ability to synchronize internal timekeeping with external cues, termed “zeitgebers”, as coined by chronobiologist Jürgen Aschoff [1]. Human physiological synchronization during the day is primarily triggered by 24 h light fluctuations [2,3]. These fluctuations convey diurnal cues to the suprachiasmatic nucleus (SCN), identified as the ‘circadian pacemaker’ located in the anterior hypothalamus [4]. The cues are transmitted via the retino-hypothalamic pathway to the SCN, synchronizing biological rhythms with the 24 h cycle and thereby impacting diverse physiological functions [5,6,7]. This synchronization contributes to daily fluctuations in both physical and cognitive performance [2,3,4,5]. Recently, there has been a growing interest in understanding the impact of CR, mainly on physical performance, in both athletic and daily life contexts [1,6,7]. In competitive situations, whether in a strenuous working environment or elite sports conditions, individuals are required to perform effectively, both physically and mentally, as many athletic activities demand a combination of motor skills, perceptual and cognitive functions [8]. Several studies support this notion, exhibiting superior skill and sport-specific performance among athletes with high cognitive performance compared to low performers [9,10,11]. Conventionally, the evaluation of functional tasks has been used as a surrogate for cognitive performance, including reaction time [12], spatial memory [13,14] and sensorimotor tasks [15,16]. Additionally, circadian variations may influence neural factors such as nerve conduction velocity, cerebral perfusion and the release of brain-derived neurotrophic factors (BDNFs) [4,5], highlighting the potential link between circadian variations in physiological functioning and altered cognitive and neuromuscular performance [17,18].

Furthermore, in the sports world, the hormonal and neuromuscular mechanisms of biorhythmic regulation, can lead to impaired cognitive function due to the onset of physical fatigue. This complex multidimensional physiological state can detrimentally affect both peripheral and central aspects of the neuromuscular system [19], depending on the mode and intensity of exercise. This trait is supported by observations that increased activation of the pre-frontal cortex (PFC) is associated with improved physical activity duration tolerance [20], possibly due to the PFC’s interaction with the basal ganglia and premotor cortex, which provide stimuli to overcome central fatigue and sustain central motor performance in the presence of inhibitory afferent signals [21,22]. Considering the role of the PFC in executive functioning, working memory and higher-order cognition, its function may be affected by neurotransmitter circadian variations [23,24]. Additionally, a state of physical fatigue resulting from exhaustive muscular activity has been shown to increase the activity of the motor cortex and the PFC, where the ability of cognition underlies a function included in both mental and physical tasks [25,26]. This finding suggests a potential connection between CR, cognition and physical performance, as they are all regulated by the frontal cortex [24]. Little is known about the ability of physiological circadian variations to modulate the susceptibility of physical and cognitive functions following a state of provoked neuromuscular fatigue.

During real-life sports events, a prolonged game duration may contribute to create fatiguing conditions due to excessive neuromuscular effort, compromising athletic performance, particularly in terms of decision making or sustained physical effort, and, as a result, impacting in-game quality and increasing injury risk [27]. A comprehensive body of research examined cognitive task-modules of visual reaction time and visual memory response following various types of exercise (e.g., handgrip dynamometer, Cycle Ergometer) [28], providing, though inconsistent, findings with some cases reporting improvements [29,30] and other reporting deteriorated results [31]. Moreover, numerous studies investigated the impact of chronotype or sleep disturbance conditions on cognition or physical performance [32,33] and also separately explored the effect of physical fatigue on cognition or physical performance in athletes [28]. However, there is currently a lack of studies evaluating the impact of both circadian variation and exercise-induced fatigue on cognitive and physical performance [34]. We argue that cognitive and physical performance indicators may be affected through intense physical activity during different times of the day, due to cyclical circadian variations in their underlying physiological components attributed by the multifaceted metabolic, hormonal and neural nature of sustaining physical performance. Thus, the aim of the present study was to investigate the effect of time-dependent circadian variations on cognitive and physical performance after fatiguing exercise. Our main hypothesis was that there will be an interaction between circadian variation and the physical fatigue state on selected cognitive and physical parameters, during three examined times of the day.

## 2. Materials and Methods

This study was approved by the Research Ethics Committee of the University of Thessaly and carried out at the Laboratory of Biomechanics and Ergonomics, ErgoMech Lab—DPESS/Physical Education and Sport Science, of University of Thessaly. The experimental design was performed in accordance with the Declaration of Helsinki. The participants mainly consisted of amateur track and field athletes, men and women with ≥4 years of training experience in athletics and team sports, with a training frequency of ≥3/week. Data were collected from 18 participants (8 males, 10 females) (Table 1) without any pre-existing musculoskeletal injuries, sleep disorders or other pathologies that may influence the findings of this study.

### 2.1. Experimental Design

Data collection took place at 09:00, 14:00 and 18:00 h in a randomized order with a minimum three-day interval between sessions to avoid exercise-induced muscle damage or mitigate the learning effect bias. Approximately 3 days before the initial session, participants underwent a familiarization session, received an overview of this study, provided written consent [35] and completed a Pittsburgh Sleep Quality Index questionnaire [36]. Participants refrained from physical exercise 24 h before testing and abstained from caffeinated beverages 2 h before testing [37]. Upon arrival at the laboratory, participants rested in a supine position for 20 min to establish the baseline resting heart rate (HRrest) following Edwards et al.’s protocol [38].

**Figure 1:**
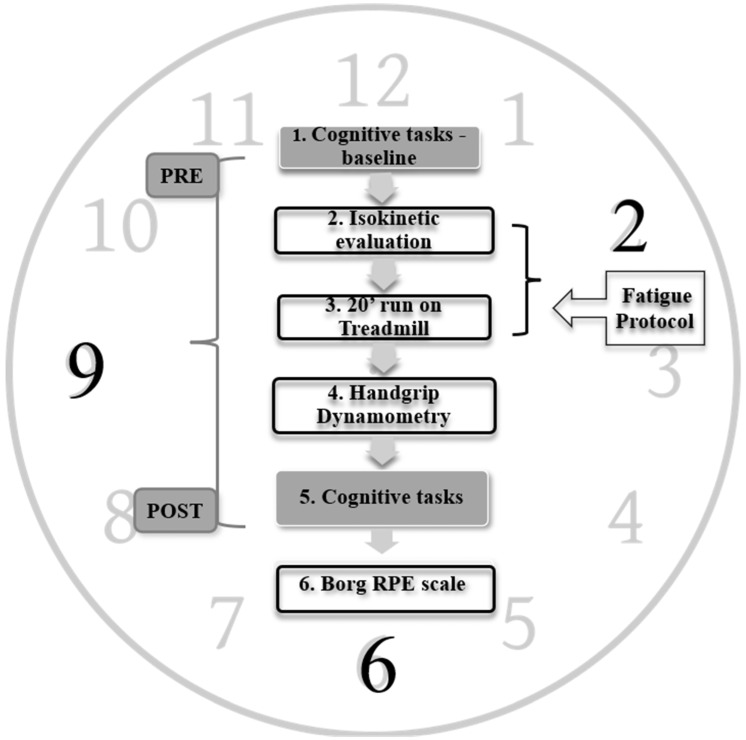
Overview of the study protocol used for the three timepoint measurements (i.e., 09:00, 14:00 and 18:00 h).

These resting heart rate recordings determined the aerobic zone of intensity during the subsequent running protocol. The fatigue experimental protocol comprised a continuous sequence of tasks designed to avoid any breaks between tasks, ensuring a continuous progression toward the primary objective of inducing a state of physical fatigue. The detailed sequence is illustrated in Figure 1, encompassing the following steps: Step 1 involved the initial baseline measurement to assess cognitive performance in a relaxed state. Subsequently, Step 2 involved isokinetic strength evaluation, followed by Step 3, which involved a 20 min running session on a treadmill. Steps 2 and 3 collectively constituted the fatigue protocol (refer to Figure 1). Following fatigue inducement, Step 4 involved assessing hand grip strength. Lastly, Step 5 encompassed the assessment of cognitive performance and rate perceived exertion (RPE) during the fatigue state. 

### 2.2. Outcomes

#### 2.2.1. Cognitive Tasks—Baseline Measurements

Initially, two distinct mental cognitive tasks were conducted using the PEBL Launcher 2.1.1 Psychological Test Battery software (Sourceforge, CA, USA). The employed tests were as follows: (a) the “Match-to-sample test”, designed to assess visual memory (VisM). This test involved memorizing a sequence of 20 matrices of squared shapes and providing accurate answers within a time limit, and (b) the “Spatial Priming test”, aimed at evaluating visual reaction time (VisRT). In this test, participants were required to click on a swiftly and continuously moving blue target, as quickly and accurately as possible within a squared frame, while ignoring the distraction of a yellow “flash” [39]. Pre-measurements of VisM and VisRT were designated as VisM1 and VisRT1, respectively. Subsequently, post-measurements of the same type were conducted at the conclusion of the fatigue protocol, employing the same methodology as the pre-fatiguing test, and denoted as VisM2 and VisRT2. 

#### 2.2.2. Isokinetic Strength Evaluation Test—Fatigue Protocol

In accordance with the study protocol (refer to Figure 1), an isokinetic strength evaluation test was incorporated into the fatigue provocation regimen. Following the completion of the mental task (refer to 2.3.1), the isokinetic test was immediately conducted. This testing protocol, as described by Tsaklis P. [40], aimed to induce muscle fatigue and employed a Cybex-Norm isokinetic dynamometer, CSMI (Salem, NH, USA). Participants started with a generalized warm-up protocol, including 10 min of low-intensity jogging on a treadmill (Runrace D140, Technogym, Cesena, Italy). This warm-up also involved dynamic stretching the major lower-limb muscle groups. Participants were then securely seated on the isokinetic dynamometer at a 90° hip flexion angle. The knee joint’s defined range of motion (ROM) was set at 0–120° with a gravitational correction at 45°. The initial protocol encompassed an 8-repetition warm-up trial, involving a concentric action of the knee extensors and flexors, with an angular velocity of 120°/s [41]. 

Subsequently, to induce fatigue, a 30-repetition protocol was executed, utilizing a concentric action of the knee extensors and flexors bilaterally at a fixed ROM of 120° and an angular velocity of 240°/s [40]. Peak torque (PT) of the knee extensors of both limbs was calculated, to evaluate the maximum strength achieved throughout the 30 repetitions. Furthermore, the fatigue index (PTFI) was computed based on the percentage of PT loss over the total attempt duration [40]. A 2 min rest period was implemented between the warm-up and fatigue protocol. The fatigue protocol was conducted randomly on the dominant/non-dominant limb and then repeated on the contralateral side after 3 min of recovery. The dominant leg was determined by asking the participant’s preferred limb when kicking a ball [42].

#### 2.2.3. Running Fatigue Protocol

Next, participants engaged in a 20 min treadmill run (Runrace D140, Technogym, Cesena, Italy) in accordance with the outlined design (see Figure 1). Heart rate monitoring, facilitated by a Polar T31 heart rate monitor (Kempele, Finland), was employed to track participants’ heart rates, ensuring the effective monitoring of activity intensity. Utilizing the initial HRrest measured at the beginning of the experimental procedure, a medium intensity at 60% and a high intensity at 84% of the maximum heart rate (HR_max_) were calculated to define the aerobic zone. During the initial 5 min of the test, participants aimed to achieve and keep at 60% of their HR_max_, followed by the subsequent 15 min performed at 84% of their HR_max_ [43]. This approach was designed to maintain participants within 84% of their HR_max_ for the majority of the 15 min duration, thereby inducing the intended fatigue state.

#### 2.2.4. Hand Grip Dynamometry

Subsequently, a hand grip strength test was conducted using the K-Force Grip^®^ 2016 (Kinvent, Monpellier, France). The participants assumed a comfortable seated position, with elbows flexed at 90° and supported by a table. Forearm and wrist positioning were adjusted to individual neutral positions [44]. Three attempts were performed with the dominant hand, and the best attempt was calculated. Each trial comprised a single maximal isometric contraction lasting 10 s of the dominant hand, with a 60 s rest period between attempts. The rate force development (RFD) was computed based on the values obtained from the K-Force Grip^®^ 2016 software, from the dominant hand during the 10 s trial [45]. Additionally, the fatigue index of the dominant hand (DynaFdh) was calculated, reflecting the decrement of isometric strength over the entire duration of the attempt. 

#### 2.2.5. Cognitive Tasks—Post Fatigue Protocol and Perceived Exertion Scale

Finally, the participants completed the testing protocol by performing the cognitive tasks post fatigue measurements of VisM (VisM2) and VisRT (VisRT2) [39]. Lastly, the participants were asked to visually indicate their subjective fatigue level using a CR-10 Borg rating of perceived exertion on a scale from 0 to 10 [18,46].

### 2.3. Statistics and Data Analysis

The sample size was determined using an a priori power analysis conducted with G*Power V 3.1.9.7 software from Heinrich-Heine-Universität, Düsseldorf, Germany. The following parameters were used within a repeated-measure ANOVA design: effect size (f) = 0.28, significance level (α) = 0.05, power = 0.80 and correlation between repeated measures (r) = 0.50. The initial power calculation indicated a sample size requirement of 15. To account for a projected ≈10% dropout rate, 19 individuals initially recruited to participate voluntarily in this study, with 18 participants completing the experimental procedure. Further statistical analyses were executed in SPSS 22.0 (IBM Inc., Chicago, IL, USA). Initially, parametric assumptions were examined by accessing data normality with the Shapiro–Wilk test. A two-way (2*3) (fatigue*time of day) repeated ANOVA on both factors was performed to evaluate the effect of the induced fatigue protocol and CR on mental cognition tasks (VisRT and VisM).

A post hoc analysis of significant interactions and main effects were further investigated using a Bonferroni adjustment. Additionally, a one-way repeated ANOVA was performed to evaluate the CR effect comparing the mean values of the Borg scale, PT, PTFI, RFD and DynaFdh measurements between the three different times of the day. A Pearson correlation coefficient analysis was conducted on all examined parameters to evaluate their correlations. Data, presented in tables as mean and standard deviations values (M ± SD), were considered significant at *p* < 0.05.

## 3. Results

### 3.1. Measures of Cognitive Performance 

A repeated ANOVA 2*3 did not show an interaction between the examined factors for VisRT (F2.22 = 0.181, *p* = 0.869) and VisM (F2.34 = 0.111, *p* = 0.895). However, a main effect of the fatigue factor (F1.11 = 15,175, *p* = 0.002) was observed. Specifically, a decrease in the parameter of VisRT was noted across all three examined timepoint measurements, suggesting an influence of the induced fatigue state on the VisRT performance. Additionally, the pre and post assessments of the VisM test exhibited non-statistical significance (F1.17 = 0.459, *p* = 0.507), suggesting no effect of the induced fatigue state on the VisM performance in the three timepoint measurements (Table 2).

### 3.2. Physical Parameters

A one-way analysis of variance did not reveal a CR effect and differences between the three timepoint measurements of the day on Borg (F2,34 = 0.668 *p* = 0.519), RFD (F2,32 = 0.342 *p* = 0.713) and DynaFdh (F2,32 = 0.311 *p* = 0.735) post fatigue measurements. However, PT (F2,30 = 4.62 *p* = 0.018), and the PTFI (F2,26 = 6.171 *p* = 0.006) showed a significant difference between the three different times of the day. Specifically, there was a significant difference between PT_9 (90.6 ± 28.1) and PT_18 (98.7 ± 31.8), where PT_18 (evening measurement) revealed a higher value than PT_9 (morning measurement) (*p* = 0.059). No other pairwise comparisons achieved significance. Additionally, there was a significant difference between PTFI_9 (36.3 ± 1.8) and PTFI_18 (41.9 ± 1.3), where PowerF_18 (evening measurement) revealed a higher value than PTFI_9 (morning measurement) (*p* = 0.012). No other pairwise comparisons achieved significance (Table 3). 

### 3.3. Correlation Analysis

There was a significant positive correlation between VisRT2_18 and DynaFdh_18 (r = 0.581, *p* < 0.01), suggesting that, as fatigue increased, cognition testing score increased as well. Additionally, a positive correlation was found between PT_14 and RFD_14 (r = 0.613, *p* < 0.01), as well as PT_18 and RFD_18 (r = 0.599, *p* < 0.01), indicating that both PT and RFD increase equally on the same time of the day (Table 4).

## 4. Discussion

The existing literature has predominantly focused on the correlation between circadian variations and physical performance, revealing a range of diurnal alterations [2]. However, minimal attention has been devoted to circadian variations in cognitive performance, particularly in the context of physical fatigue conditions. This study aimed to investigate the effects of the time of day and exercise-induced fatigue on both cognitive and physical performance. Our initial hypothesis was partially confirmed. While we found no significant interaction between circadian variation and the physical fatigue state on selected cognitive and physical parameters at the three times of the day examined, there was a significant main effect of fatigue on cognitive performance. Additionally, certain physical parameters related to strength exhibited circadian variations.

### 4.1. CR and Cognitive Performance

The current findings indicate that CR did not exert an effect on cognitive performance, specifically VisM and VisRT, even under conditions of induced physical fatigue. This observation is consistent with a study conducted by Nogueira et al. (2021), which explored circadian variations in high-intensity motor tasks using the Grooved Pegboard test. Their research revealed no performance differences, suggesting that cognitive function, in the absence of physical exertion, may not be significantly influenced by circadian rhythms [47]. This finding contrasts with the results reported by Rulleau and colleagues (2015), who previously identified circadian variations in cognitive parameters related to the temporal coupling between executed and simulated actions during motor imagery tasks in older adults. In their study, Rulleau et al. observed favorable performance during morning hours, even in the absence of physical fatigue [48]. The different findings compared to our study may be due to the absence of fatigue conditions in the Rulleau et al. study and the age difference between the study samples. Moreover, it should be noted that, due to the multifaceted nature of cognitive capacity, the literature presents a debatable stance on circadian variations. Cognition may be influenced by both endogenous factors (e.g., age, neural network malfunction) and exogenous factors (e.g., sleep deprivation, task difficulty, chronotype preference) [22,49,50]. Consequently, results may vary based on the applied protocol and potential study limitations [14]. Thus, given the heterogeneity of our study design compared to the previously mentioned investigations, a direct comparison of results may not be appropriate.

### 4.2. Physical Fatigue and Cognitive Performance

Despite the non-significant interaction of CR and fatigue in the cognition tasks, the statistical outcome indicated a main effect of fatigue on VisRT, appearing to positively influence cognitive function after the fatigue protocol was applied (Table 2). This outcome might vary in attribution, as, according to the literature, the effects of acute fatiguing exercise on cognition range from strongly positive to negative [21,51,52,53]. This variability is primarily due to the lack of standardized evaluation methods for the influence of fatiguing exercise on cognitive functioning, given the differing intensities and durations of physical activity protocols used [52,53]. Additionally, participant bias during the second, post fatigue cognitive measurement might have influenced the results. [54]. However, substantial evidence supports the notion that acute fatiguing exercise generally enhances cognitive functioning, particularly tasks involving the prefrontal cortex. For instance, studies such as Hendy et al. 2022, reveal that high-intensity aerobic exercise affects both excitability and inhibition in the upper limb motor cortex [55]. This exercise modality is also linked to increased levels of the circulating BDNF, a crucial element in neural plasticity and synaptic transmission [56]. Neuroplasticity induced by aerobic exercise, even from a single session, can alter cortical excitability and impact neural circuits significantly [57]. Furthermore, studies have reported improved cognitive performance at different times of the day related to strength training, attributed to a favorable neural metabolic environment at the neuromuscular junction [58,59]. This enhancement is primarily driven by increased blood flow, boosting cerebral cortex perfusion, and engaging BDNF regulation [17,18]. Consequently, resistance exercise modalities may enhance cognitive performance due to improved nerve conduction velocity, cerebral perfusion, and BDNF release [60,61]. Supporting this finding, the negative correlation between the VisRT2_18 variable and both RFD_18 and DynaFdh_18 suggests that, during evening measurements, higher strength development and the fatigue index in strength-related modalities correspond to improved performance on cognitive reaction time tests.

### 4.3. CR and Physical Parameters

In the present study, we examined several physical performance indicators after applying a fatigue protocol at three different times of the day. The results showed no statistically significant differences in the rate of perceived exertion (Borg), the rate of force development (RFD) and Handgrip Dynamometry Fatigue Index (DynaFdh) measurements across these timepoints. However, a notable CR effect was observed for PT and PTFI, which both demonstrated higher outcomes at 18:00 h compared to 09:00 h. These findings suggest that PT and PTFI, which are crucial elements of strength capability, are influenced by the time of day, with improved performance in the evening. This finding could be attributed to physiological variations such as body temperature and hormonal levels that peak later in the day [1]. Such circadian changes in body temperature could potentially affect nerve conduction velocity, explaining previously observed improvements for power output, coordination and reaction time performance shown in the late afternoon [62,63]. Additionally, other studies have implicated circadian variations in cortisol and its effect on body temperature and muscle performance, displaying a higher PT and RFD during evening hours compared to morning outputs, evaluated via isokinetic or handgrip dynamometry tasks [64,65].

Notably, the findings of higher strength performance during evening hours and an increased fatigue index during the same period might be explained by the increased demand on anaerobic energy systems [66], the recruitment of fast-twitch muscle fibers [23,66], the accumulation of metabolic waste products [67], and potential central nervous system alterations, [68], all of which contribute to muscle fatigue during high-intensity activities. Studies like those of Hammouda et al. [69] and Deschenes et al. [64] demonstrated significant diurnal effects, with improved the performance for PT and total work performance among the participants performing maximal resistance exercise protocols using an isokinetic dynamometer. Thus, the observed peak in PT and the PTFI in the evening indicates a direct relationship between increased power output and the simultaneous increase in fatigue development.

## 5. Conclusions

Although we found no interactions between circadian variation and the physical fatigue state on selected cognitive parameters at the three times of the day examined, there was an effect of fatigue on cognitive performance. Additionally, physical parameters related to strength exhibited circadian variations, exhibiting peak values in the evening hours. Improving our understanding of how humans perform mentally, concerning circadian variations, in both rested and physically fatigued states, could help to inform sound athletic practices, the prevention of musculoskeletal injuries and promote psycho-physiological well-being. We encourage future studies to delve deeper into biochemical markers, diverse physical fatigue protocols and neurological aspects of cognitive performance during different times of the day. Implementing these factors may allow future authors to expand upon the circadian underpinnings of cognitive performance across diverse populations.

## 6. Limitations and Future Recommendations

Although one of this study’s strengths was the low dropout rate of participants (only one person), the overall research presents several limitations. This study did not investigate biochemical markers to elucidate the behavior of cognitive and physical parameters at different times of the day after physical fatigue provocation. Additionally, the research focused on a general physical fatigue regime, whereas differentiating between central and peripheral fatigue protocols might have revealed different results. A future recommendation could be to record brain activity, to gain detailed insights into the neurological aspects of cognitive performance following physical fatigue provocation at various times of the day.

## Figures and Tables

**Table 1:** Anthropometrics and baseline parameters used to set up the fatigue protocol based on the HR_max_ aerobic zone at the three timepoint measurements.

Characteristics	09:00 h (Morning) (N = 18)	14:00 h (Afternoon) (N = 18)	18:00 h (Evening) (N = 18)
Age (years)	24.7 ± 4.5	24.7 ± 4.5	24.7 ± 4.5
Body mass (kg)	65.4 ± 8.9	65.4 ± 8.9	65.4 ± 8.9
HR rest (bpm)	68.3 ± 5.2	68.9 ± 7.5	67 ± 6.3
60% of HR_max_ (bpm)	141.4 ± 4.5	141.7 ± 3.9	141.0 ± 3.5
84% of HR_max_ (bpm)	170.7 ± 3.1	171.3 ± 3.5	170.6 ± 2.9

Data are expressed as mean values and standard deviation (M ± SD). Abbreviations: HR rest: heart rate measured in resting state; 60% of HR_max_: 60% of the maximum heart rate within the aerobic zone; 84% of HR_max_: 84% of the maximum heart rate within the aerobic zone; and bpm: beats per minute.

**Table 2:** Pre and post fatigue cognitive performance across the three timepoint measurements.

	09:00 (Morning)		14:00 (Afternoon)		18:00 (Evening)		F_df_			*p* Value		
	Pre	Post	Pre	Post	Pre	Post	Fatigue	TofDay	Fatigue * TofDay	Fatigue	TofDay	Fatigue * TofDay
VisRT	1.70 (0.23)	1.62 (0.13)	1.71 (0.24)	1.60 (0.14)	1.70 (0.26)	1.57 (0.16)	F_1.11 =_ 15.17	F_2.22_ = 0.94	F_2.22 =_ 0.18	*p* < 0.01 *	*p* > 0.05	*p* > 0.05
VisM	2.89 (1.81)	3.17 (1.91)	2.83 (1.50)	2.89 (1.60)	2.94 (2.18)	3.33 (2.27)	F_1.17_ = 0.6	F_2.34_ = 0.26	F_2.34_ = 0.11	*p* > 0.05	*p* > 0.05	*p* > 0.05

Data represent mean values ± standard deviation (SD). Bold values indicate a significant difference from baseline (*p* < 0.05). * indicates statistical significance of *p* < 0.05, df: degrees of freedom, VisRT: visual reaction time, VisM: visual memory, TofDay: time of the day, fatigue * TofDay: interaction between fatigue and time of the day factors.

**Table 3:** Physical parameters for the three timepoint measurements, after inducement of fatigue state.

Parameter	09:00 (Morning)	14:00 (Afternoon)	18:00 (Evening)	F_df_	*p* Value
Borg	4.80 (1.20)	5.20 (1.00)	5.20 (1.60)	F_2.34_ = 0.67	0.519
RFD	137.30 (76.70)	161.20 (109.40)	145.8 (112.40)	F_2.32_ = 0.34	0.713
DynaFdh	0.86 (0.22)	1.00 (0.21)	0.99 (0.20)	F_2.32_ = 0.31	0.735
PT	90.60 (28.10)	96.10 (33.80)	98.70 (31.80)	F_2.30_ = 4.62	0.018 *
PTFI	36.30 (6.90)	40.70 (5.20)	41.90 (5.10)	F_2.26_ = 6.17	0.006 *

Data represent mean values ± standard deviation (SD), F-values, df: degrees of freedom. Bolded values indicate a significant difference between the different times of the day (*p* < 0.05). * indicates statistical significance of *p* < 0.05. Borg: rate of perceived exertion; RFD: rate of force development; DynaFdh: handgrip dynamometry fatigue index of dominant hand; PT: peak torque of isokinetic evaluation; and PTFI: peak torque fatigue index of isokinetic evaluation.

**Table 4:** Correlation between cognitive and physical parameters, of the three different times of the day.

Parameter	VisRT2_9	VisRT2_14	VisRT2_18	VisM2_9	VisM2_14	VisM2_18	PT_9	PT_14	PT_18	PTFI_9	PTFI_14	PTFI_18	RFD_9	RFD_14	RFD_18	DynaFdh_9	DynaFdh__14	DynaFdh_18
VisRT2_9	1	0.324	0.000	0,010	0.071	0.078	0.115	0.183	0.271	−0.164	0.041	0.185	−0.220	−0.012	−0.002	0.270	0.070	−0.229
VisRT2_14	0.324	1	0.112	−0.133	0.203	0.028	0.544	0.421	0.385	0.950	0,168	0.879	0.656	0.491	0.894	0.873	0.216	0.916
VisRT2_18	0.000	0.112	1	0.606	0.129	0.077	0.251	0.421	0.681	0.343	0.309	0.083	0.334	0.329	−0.547 *	0.986	0.193	−0.581 **
VisM2_9	0.010	−0.133	0.130	1	0.083	0.297	0.154	0.129	0.028	0.550	0.295	−0.150	−0.204	0.407	0.059	−0.143	0.191	−0.021
VisM2_14	0.071	0.203	0.372	0.083	1	0.446	−0.176	−0.205	−0.141	−0.090	−0.286	−0.192	−0.369	−0.156	−0.341	0.045	0.364	0.454
VisM2_18	0.078	0.028	0.427	0.297	0.446	1	0.229	0.182	0.138	0.066	−0.027	−0.289	−0.470 *	0.058	−0.199	0.183	0.074	0.318
PT_9	0.115	−0.164	0.305	0.154	−0.176	0.229	1	0.964 **	0.921 **	−0.036	−0.175	−0.086	0.141	0.717 **	0.507 *	0.322	0.047	0.170
PT_14	0.183	−0.216	0.216	0.129	−0.205	0.182	0.964 **	1	0.955 **	−0.075	−0.048	−0.090	0.137	0.613 **	0.634 **	0.285	0.058	0.160
PT_18	0.271	−0.233	0.112	0.028	−0.141	0.138	0.921 **	0.955 **	1	−0.114	−0.116	−0.007	0.290	0.520 *	0.599 **	0.313	0.005	0.080
PTFI_9	−0.164	−0.018	−0.274	0.550 *	−0.090	0.066	−0.036	−0.075	−0.114	1	0.446	0.527 *	−0.004	0.206	−0.090	0.213	0.014	−0.010
PTFI_14	0.041	−0.390	−0.293	−0.295	−0.286	−0.027	−0.175	−0.048	−0.116	0.446	1	0.246	0.020	−0.009	0.022	0.005	0.240	−0.101
PTFI_18	0.185	0.045	−0.475	−0.150	−0.192	−0.289	−0.086	−0.090	−0.007	0.527 *	0.246	1	−0.050	−0.089	−0.244	0.098	−0.255	−0.420
RFD_9	−0.220	−0.116	−0.250	−0.204	−0.369	−0.470 *	0.141	0.137	0.290	−0.004	0.020	−0.050	1	0.207	0.324	0.068	0.005	−0.149
RFD_14	−0.012	−0.179	0.252	0.407	−0.156	0.058	0.717 **	0.613 **	0.520 *	0.206	−0.009	−0.089	0.207	1	0.311	0.079	0.194	0.052
RFD_18	−0.002	−0.035	−0.547 *	0.059	−0.341	−0.199	0.507 *	0.634 **	0.599 **	−0.090	0.022	−0.244	0.342	0.311	1	0.139	0.229	0.578 *
DynaFdh_9	−0.270	−0.042	0.004	−0.143	0.045	0.183	0.322	0.285	0.313	0.213	0.005	0.098	0.068	0.079	0.139	1	0.298	0.417
DynaFdh_14	0.070	0.316	0.332	−0.191	0.364	0.074	0.047	0.058	0.005	0.014	0.240	−0.255	0.005	0.194	0.229	0.298	1	0.731 **
DynaFdh_18	−0.229	0.028	−0.581 **	−0.0021	0.454	0.0318	0.170	0.160	0.080	−0.010	−0.101	−0.420	−0.149	0.052	0.578 *	0.417	0.731 **	1

Data represent values of Pearson’s r correlation, * Correlation is significant at the 0.05 level (2-tailed), ** Correlation is significant at the 0.01 level (2-tailed). VisRT 2_9: visual reaction time post fatigue test at 09:00 h, VisRT 2_14: visual reaction time post fatigue test at 14:00 h, VisRT 2_18: visual reaction time post fatigue test at 18:00 h, VisM 2_9: visual memory post fatigue test at 09:00 h, VisM 2_14: visual memory post fatigue test at 14:00 h, VisM 2_18: visual memory post fatigue test at 18:00 h, PT_9: peak torque at 09:00 h, PT_14: peak torque at 14:00 h, PT_18: peak torque at 18:00 h, PTFI_9: peak torque fatigue index at 09:00 h, PTFI_14: peak torque fatigue index at 14:00 h, PTFI_18: peak torque fatigue index at 18:00 h, RFD_9: rate of force development at 09:00 h, RFD_14: rate of force development at 14:00 h, RFD_18: rate of force development at 18:00 h, DynaFdh_9: handgrip dynamometry fatigue index of dominant hand at 09:00 h, DynaFdh_14: handgrip dynamometry fatigue index of dominant hand at 14:00 h, and DynaFdh_18: handgrip dynamometry fatigue index of dominant hand at 18:00 h.

## Data Availability

The raw data supporting the conclusions of this article will be made available on request by the first author.
